# The Best of Two Worlds: IRT-Enhanced Automated Essay Interpretable Scoring

**DOI:** 10.3390/bs16040542

**Published:** 2026-04-06

**Authors:** Wei Xia, Jin Wu, Jiarui Yu, Chanjin Zheng

**Affiliations:** 1 Department of Educational Psychology, Faculty of Education, East China Normal University, Shanghai 200062, China; 2 Shanghai Institute of Artificial Intelligence for Education, East China Normal University, Shanghai 200062, China; 3 School of Computer Science and Technology, Faculty of Information, East China Normal University, Shanghai 200062, China

**Keywords:** Automated Essay Scoring, Item Response Theory, Bidirectional Encoder Representations from Transformers, Generalized Partial Credit Model

## Abstract

The Automated Essay Scoring (AES) systems confront two fundamental challenges: opaque “black-box” decision-making that limits educator trust, and insufficient validation across linguistically diverse educational contexts. This study proposes IRT-AESF, an innovative framework that bridges educational measurement theory and artificial intelligence by integrating item response theory (IRT) with deep learning. The framework generates three theoretically grounded psychometric parameters: student ability, item difficulty, and item discrimination, which provide transparent and interpretable explanations for scoring decisions. We rigorously evaluated IRT-AESF through 5-fold cross-validation on three large-scale datasets comprising 41,328 authentic essays from English and Chinese educational settings, including both classroom assessments and high-stakes examinations. Results demonstrate statistically significant improvements over competitive baseline models, achieving an 8.4% relative increase in quadratic weighted kappa while maintaining robust cross-lingual performance. This research advances the development of transparent, trustworthy automated assessment systems that deliver not only scores but meaningful diagnostic insights for educational practice.

## 1. Introduction

Assessing writing proficiency is fundamental to students’ academic development and serves as a cornerstone of educational evaluation (Srikandi, 2019). In this domain, scoring by human experts remains the benchmark for validity. This approach is grounded in rigorous psychometric principles and relies on standardized protocols such as rater selection, training, and multi-rater blind scoring. Moreover, sophisticated measurement models, including generalizability theory (G-Theory) or item response theory (IRT), are employed to ensure its reliability and fairness (Engelhard, 1992; Reise & Waller, 2009).

Despite its high validity, this manual method presents significant logistical challenges. The process is both time-consuming and resource-intensive, creating a critical bottleneck for large-scale assessments. Consequently, the delivery of timely, formative feedback to students is often delayed. This situation reveals an inherent trade-off between maintaining assessment quality and achieving operational scalability.

To address this scalability challenge, automated essay scoring (AES) has emerged as a promising solution. AES has progressed significantly since its inception with Page’s (1966) Project Essay Grade (PEG). Contemporary systems based on large language models (LLMs) are now demonstrating impressive scoring accuracy, often rivaling that of human raters (Li & Liu, 2024; Mansour et al., 2024; Pack et al., 2024; Yavuz et al., 2024). This advancement, however, has introduced two pressing challenges for the field of educational technology.

First, despite significant advancements, most current AES research remains heavily English-centric, leaving a substantial gap in validation across linguistically diverse contexts. Non-English datasets, such as Chinese, which feature unique logographic structures and distinct syntactic patterns, are markedly underrepresented in existing literature. This lack of cross-linguistic evidence limits the global generalizability of AES systems and underscores the urgency of our study, which validates the IRT-AESF framework across both English and large-scale Chinese datasets.

Second, beyond its evaluative function, the role of AES as a cornerstone of the formative feedback ecosystem is increasingly recognized. Recent empirical evidence from a large-scale randomized controlled trial (n=918) demonstrates that generative AI-based feedback can yield statistically significant improvements in student writing quality while simultaneously fostering higher levels of task engagement and eliciting nuanced emotional experiences during the revision process (Chan et al., 2024). However, the pedagogical efficacy of such feedback remains tethered to the underlying reliability and transparency of the scoring logic. Without an “interpretable-by-design” mechanism to anchor these AI-generated insights, there is a risk of providing feedback that, while engaging, lacks the psychometric rigor needed for precise instructional calibration. This underscores the necessity of frameworks like IRT-AESF, which provide the latent ability estimates (θ) and threshold structures (β) required to underwrite more trustworthy and pedagogically targeted feedback workflows.

The development of automated essay scoring has undergone a significant evolution. Foundational operational systems, such as ETS’s e-rater and Pearson’s Intelligent Essay Assessor, established the early paradigm by relying on handcrafted linguistic features. While these systems provided initial scalability, recent advancements have largely shifted from feature-engineered systems to deep learning frameworks to better capture complex semantic representations. Unlike previous attempts to integrate IRT into AES, which were often constrained by narrow model compatibility or limited to specific neural architectures, our proposed IRT-AESF framework introduces a model-agnostic “plug-in” design. By decoupling the psychometric GPCM layer from the primary encoder, our approach overcomes the limitations of prior research, ensuring seamless integration with diverse backbones ranging from state-of-the-art transformers to traditional recurrent networks.

The growing complexity of these models has led to an “interpretability problem”. The models often function as opaque “black boxes”, which makes it difficult for educators to understand or trust their scoring rationale (Wang & Hu, 2021; Y. Kumar et al., 2023). In addition, a significant “generalization problem” persists. Much of the existing AES research is validated on a limited set of English-language, high-stakes assessment corpora (e.g., ASAP-AES; Mathias & Bhattacharyya, 2018). This narrow focus fails to represent the linguistic and contextual diversity of authentic educational settings. As a result, the robustness of these models in real-world, multi-context scenarios remains largely unverified.

This imbalance raises critical concerns regarding cross-linguistic robustness and construct validity in high-stakes assessment environments. Therefore, validating interpretable AES frameworks in multilingual contexts is not merely a supplementary extension but a necessary step toward broader architectural applicability.

To address these dual challenges of interpretability and generalizability, this work proposes and evaluates the item response theory-enhanced AES framework (IRT-AESF). This framework is designed to bridge the gap between the predictive power of modern AI and the explanatory rigor of established educational measurement. By deeply integrating an IRT module into the AES pipeline, the IRT-AESF moves beyond simple score prediction. The framework enhances interpretability by providing interpretable designs of scoring decisions (Gilpin et al., 2018; Arrieta et al., 2019; Guidotti et al., 2019; Murdoch et al., 2019). It also enriches the assessment output with psychometrically meaningful diagnostic parameters, such as item (prompt^1^) difficulty and discrimination. This integration ensures the framework is not only accurate but also transparent and educationally informative, positioning it for robust performance across diverse linguistic and instructional contexts.

This study makes the following primary contributions to educational technology research and development:

(1) The proposal of an interpretable and generalizable framework, IRT-AESF, that integrates educational measurement theory with diverse AES technologies to mitigate the “black-box” problem and enhance scoring transparency. The framework provides prompt-internal diagnostic parameters, such as relative student writing ability and prompt-specific threshold structures, ensuring internally coherent parameters that enhance within-prompt scoring consistency.
(2) A demonstration of the framework’s versatility by implementing and evaluating it with two distinct classes of state-of-the-art models: a deep neural network and a pretrained language model.
(3) Rigorous empirical validation of the framework’s effectiveness and generalizability using three large-scale, multilingual, and multi-context datasets from authentic educational assessments.

The remainder of this paper is structured as follows: Section 2 reviews related work in AES and interpretability, Section 3 introduces the proposed framework, Section 4 details the empirical studies, and Section 5 discusses the findings and concludes the paper.

## 2. Literature Review

This literature review provides the rationale for the proposed framework. First, it delineates the foundational principles of rigorous, interpretable essay scoring from the field of educational measurement. Second, it traces the technological evolution of automated essay scoring (AES) and highlights the emergent challenges of interpretability and generalizability. Finally, the review examines prior work that integrates measurement theory with AES technology. This examination identifies the specific research gaps that the present study aims to address.

### 2.1. Foundational Principles of Rigorous Essay Scoring

Early operational AES systems, notably e-rater and the Intelligent Essay Assessor (IEA), primarily utilized natural language processing techniques to extract surface-level and syntactic features for scoring. The psychometric integrity of large-scale manual essay scoring relies on two key components: rigorous operational procedures and the statistical models that validate them.

#### 2.1.1. Operational Procedures of Manual Essay Scoring

**Figure 1:**
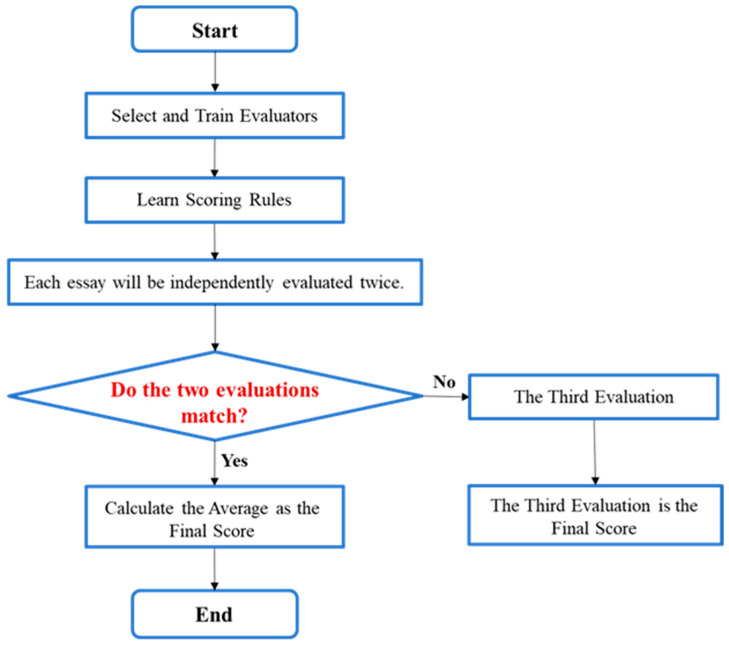
Large-Scale Manual Essay Scoring Workflow.

In educational assessment, a multi-stage process ensures the credibility of manual scoring. The process begins with the careful selection of qualified raters. These raters then undergo systematic training using specific scoring rubrics (Moskal & Leydens, 2000; Andrade et al., 2008; Rezaei & Lovorn, 2010; Panadero & Jonsson, 2013). To promote fairness, each essay is independently evaluated by at least two raters in a double-blind scoring system. A critical final step is the verification of rater consistency. When the initial scores are consistent, their average is taken as the final score. If a significant discrepancy occurs, a third, senior rater adjudicates to resolve the difference (Powers et al., 1998; Rezaei & Lovorn, 2010; Shermis, 2014; Campion et al., 2016). Collectively, these procedures ensure that scoring is a systematic, evidence-based practice rather than an arbitrary judgment. The standardized workflow for large-scale manual essay scoring is illustrated in Figure 1.

#### 2.1.2. Important Models in Educational Measurement

These operational procedures are grounded in the principles of educational measurement. Key measurement models, such as generalizability theory (Shavelson & Webb, 1981; Shavelson et al., 1992; Brennan, 1992, 2010; Briesch et al., 2014) and item response theory (Samejima, 1968; Baker, 2001; Embretson & Reise, 2013), are central to establishing the psychometric soundness of these scores. IRT offers a powerful statistical framework for connecting a student’s latent ability (e.g., writing proficiency) to their observed scores. A key advantage of IRT is its interpretability. The model yields meaningful parameters, including student ability θ, item discrimination $\alpha_{j}$, and item difficulty $\beta_{j}$.

For polytomous (i.e., multi-category) scoring common in essay assessment, the generalized partial credit model (GPCM) is a widely used and robust IRT model (Muraki, 1992, 1993; Muraki & Muraki, 2016). The mathematical formulation of GPCM is as follows:

(1) $$P_{ijk}\left(\theta \right)=\frac{exp[\sum_{m=1}^{k}D\alpha_{j}(\theta -\beta_{jm})]}{\sum_{l=1}^{K}exp[\sum_{m=1}^{l}D\alpha_{j}(\theta -\beta_{jm})]}$$

where i denotes the student, j the essay prompt, k the score category (ranging from 1 to K), D a constant usually set to 1.7, θ the student’s latent ability, $\alpha_{j}$ the discrimination parameter of the essay prompt j, $\beta_{j}$ the difficulty of moving from step m−1 to step m in the essay item j, and $P_{ijk}$ the probability that the student i selects score category k for item j.

From a data science perspective, the core mathematical form of the GPCM is analogous to a softmax function that incorporates a latent variable. However, this theory-guided approach requires intensive manual labor, which makes the scoring process slow and costly. These logistical constraints limit its scalability and highlight the need for automated solutions.

### 2.2. The Technological Response: Evolution and Challenges of AES

Automated essay scoring (AES) was developed by researchers across disciplines, including psychology and computer science, to address the efficiency limitations of manual scoring. This technology has evolved over time, leading to improved performance. However, this evolution has also introduced new and significant challenges.

#### 2.2.1. The Evolution of AES Models

Since its inception with the PEG system (Page, 1966), AES has progressed through several paradigms. Early approaches, based on heuristics (Persing et al., 2010) and statistical machine learning (McNamara et al., 2015; Ramalingam et al., 2018), offered a degree of interpretability. However, they had a limited capacity to capture deep semantic meaning, which restricted their scoring accuracy.

The modern era of AES can be characterized by three successive technological waves. A summary of these development paradigms, including their key technologies and respective trade-offs, is provided in Table 1. The first wave, deep neural networks (DNNs), improved automated feature extraction. However, these models are inherently “black boxes” and often struggle to capture long-range contextual dependencies in essays (Hussein et al., 2020; V. Kumar & Boulanger, 2020; Yuan et al., 2020; Uto et al., 2020; Uto, 2021; Tashu et al., 2022). The second wave, leveraging pre-trained language models (PLMs) like BERT, substantially improved scoring consistency by enhancing contextual semantics recognition (Yang et al., 2020; Ormerod et al., 2021; Wang et al., 2022; Mizumoto & Eguchi, 2023). However, the increased parameter counts and complexity of PLMs exacerbated the “black-box” problem. Most recently, the third wave of generative large language models (LLMs), such as those in the GPT series, has introduced new possibilities for AES (Mansour et al., 2024; Pack et al., 2024). Yet, these large-scale models pose even greater challenges to interpretability. This evolutionary trend highlights a critical trade-off: as model performance increases, model transparency decreases. Consequently, the “black-box” issue has become a central challenge in the field (Yang et al., 2020; Wangkriangkri et al., 2020; Ormerod et al., 2021; Wang et al., 2022; Mizumoto & Eguchi, 2023).

While generative LLMs have significantly improved the quality of automated feedback, they often struggle with the “black-box” nature of score derivation and consistency across diverse prompts. The proposed IRT-AESF framework addresses these limitations by providing a theoretically grounded psychometric layer that can be integrated with various encoders, including LLMs. This integration allows for high-resolution scoring and latent trait estimation while maintaining computational efficiency compared to prompt-based LLM grading.

#### 2.2.2. The Interpretability Challenge

The performance gains of modern AES have been achieved at the expense of transparency. This “black-box” nature contrasts sharply with the theory-driven approach of educational measurement outlined in Section 2.1. Within machine learning, interpretability can be categorized by its timing relative to model training and its scope of explanation (Arrieta et al., 2019; Murdoch et al., 2019). These dimensions and their application to our proposed framework are summarized in Table 2. Transparent models are interpretable by design due to their simple structure (e.g., linear regression). However, their simplicity often limits their performance on complex tasks like AES. Consequently, the predominant approach involves applying complex models first and subsequently providing post hoc explanations. Post hoc methods analyze a trained model from the outside to explain its predictions without altering it (Guidotti et al., 2019). These explanations can be global (explaining the model’s overall behavior) or local (explaining a single prediction). In AES, many existing post hoc techniques are designed for system developers and produce outputs that are not readily useful for educators and students. This work directly confronts this challenge. Rather than generating low-level technical outputs, our framework integrates IRT to provide a post hoc, global explanation that yields high-level, educationally meaningful, and immediately interpretable parameters such as student ability, prompt difficulty, and prompt discrimination.

#### 2.2.3. The Generalization Challenge

Beyond interpretability, AES faces a significant generalization challenge. To understand the empirical landscape, this work conducted a literature search in the Scopus database, identifying 125 papers on the AES task over the past 10 years, excluding related but distinct tasks like grammatical error correction or rhetoric identification. The analysis of these papers from three perspectives reveals a heavy reliance on limited and homogeneous data.

**Figure 2:**
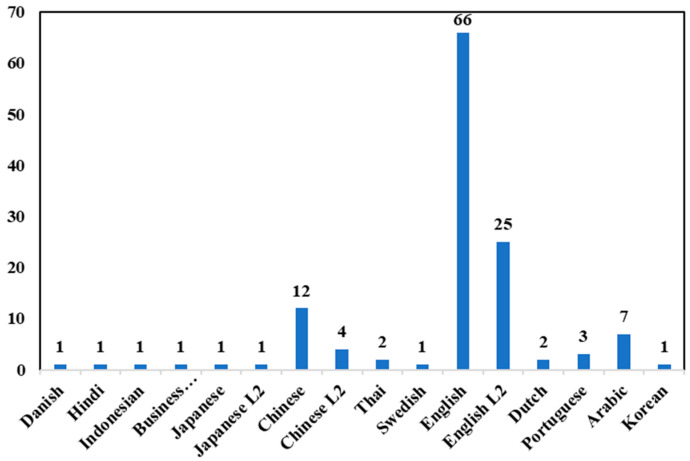
Usage of Different Language Datasets.

**Figure 3:**
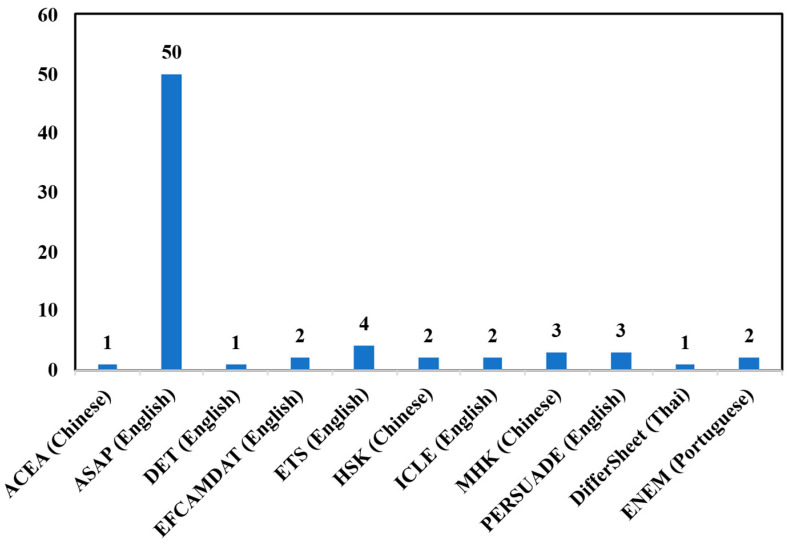
Application of Different Languages in Public Datasets.

**Figure 4:**
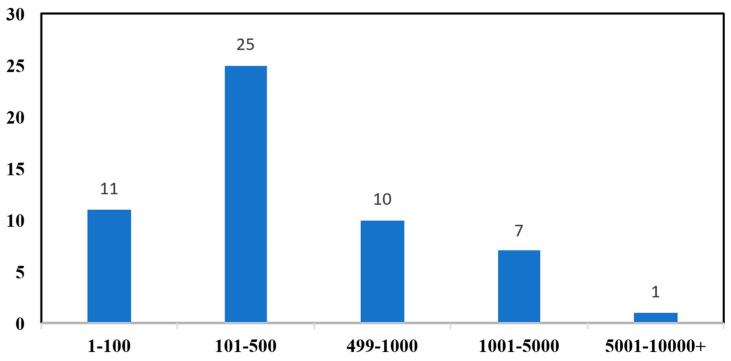
Scale of Experimental Data in Self-Collected Datasets.

(1) Linguistic Context: Research is overwhelmingly concentrated on English-language essays, with few studies in other languages like Chinese, where large-scale assessment is also prevalent. The distribution of research across different language contexts is illustrated in Figure 2.

(2) Dataset Variety: A majority of studies (57%) use a single public dataset—primarily the ASAP-AES corpus (Mathias & Bhattacharyya, 2018). This limits the validation of model performance across different writing prompts, genres, and educational levels. Figure 3 provides a breakdown of how different languages are represented across these public datasets.

(3) Data Scale: Among studies using self-collected data, over two-thirds use fewer than 500 essays. Such small datasets are insufficient for validating models intended for large-scale deployment. The distribution of experimental data scales for studies using self-collected datasets is shown in Figure 4.

Collectively, these issues highlight a critical data bottleneck in the field. The scarcity of non-English corpora, the limited use of diverse public datasets, and the small scale of private datasets mean that the generalizability of most AES models in authentic educational settings remains largely unsubstantiated.

To address the inherent ordinal nature of essay scores, recent studies have introduced modern ordinal modeling baselines, such as Consistent Rank Logits (CORAL). While these generic ordinal regression methods successfully enforce rank consistency across score categories, they typically apply a universal ordinal assumption across all tasks. In contrast, our integration of the Generalized Partial Credit Model (GPCM) goes beyond simple ordinality by explicitly modeling item-specific difficulty and discrimination parameters, thereby capturing the unique psychometric properties of each individual prompt.

### 2.3. Bridging the Divide: Previous Works on IRT-AES Integrations and the Remaining Research Gaps

To address the challenges of interpretability and generalization, some studies have begun to integrate the psychometric principles from Section 2.1 with the machine learning models from Section 2.2. This integrative approach has shown promise in several related fields within educational AI. For instance, in cognitive diagnosis, the DIRT framework employs a deep neural network to extract student and item features. An IRT model then uses these features to provide interpretable diagnoses of knowledge mastery (Cheng et al., 2019). Similarly, in the field of knowledge tracing, the Deep-IRT model integrates a neural network with IRT to model student learning trajectories. The IRT component provides interpretable parameters for student ability and item difficulty (Yeung, 2019). Collectively, these studies demonstrate the viability of combining deep learning with IRT to enhance model interpretability.

Within the field of AES, researchers have also begun to explore such integrations. A significant line of research by Uto has applied IRT to address psychometric challenges in AES. Their work focuses on modeling and correcting for rater effects and linking scores across different essay datasets (Uto & Okano, 2022; Uto et al., 2023; Uto & Aramaki, 2024). While the present study shares a similar conceptual foundation, its research objectives differ. This work’s focus is on enhancing the post hoc interpretability of the core scoring model, rather than on addressing rater effects or data linking. The work by Shibata and Uto (2022) is most directly relevant to our research. They incorporated an IRT model into a DNN-based AES to facilitate multidimensional trait scoring, demonstrating the potential for integrating IRT directly into the AES process.

Prior research by Uto (2019) and Uto et al. (2020) demonstrated the utility of IRT in addressing rater effects and establishing score equivalence across different assessment forms. Our study diverges from this focus by prioritizing the post hoc interpretability and diagnostic value of the neural scoring architecture. By embedding the GPCM layer directly into the deep learning pipeline, we move beyond administrative score linking toward an ‘interpretable-by-design’ mechanism that clarifies why a specific score was assigned based on prompt-specific psychometric parameters.

Despite these promising developments, several research gaps remain. This study aims to address these gaps by making three key contributions. First, we move beyond previous integration approaches to propose a comprehensive, interpretable framework. This framework systematically combines principles from educational measurement with methods from machine learning. Second, we expand the technological scope of this integration. While previous studies focused primarily on deep neural network architectures, the proposed framework is designed for a broader range of contemporary “black-box” models, including pre-trained language models. Third, we address the challenge of generalization by validating the framework on diverse, large-scale datasets from real-world settings. Prior work in this area has often relied on the public ASAP dataset. This study extends the evaluation to include two additional large-scale Chinese essay datasets, one from a classroom context and another from a high-stakes assessment.

## 3. Methodology

This section details the methodology used to develop and validate the proposed framework. We first define the AES task from an educational measurement perspective. We then explain the rationale behind our choice of model components, describe the overall architecture, and specify the learning objective for model training.

### 3.1. The IRT-AESF Scoring Framework

In this work, we conceptualize the AES task as a multi-class classification problem, where the goal is to assign an essay to one of several predefined score categories. For any given essay prompt j, the dataset consists of a collection of essays ($X=\{{x_{i}\}}_{i=1}^{n}$) and their corresponding scores ($Y={\left\{y_{i}\right\}}_{i=1}^{n}$) awarded by expert human raters. Each score $y_{j}\in \{0,1,2\ldots ,C-1\}$, where C is the total number of score categories. The framework is designed with a dual purpose. The primary objective is educational: to learn a function that links an essay $x_{i}$ to a predicted score ($S_{i}$) that is consistent with human judgment. The secondary, and equally important, goal is to enhance the model’s interpretability. It achieves this by estimating two key sets of psychometric parameters: (1) each student’s underlying writing ability ${(\theta }_{i}$), and (2) the assessment characteristics of the prompt itself, namely its discrimination ${(\alpha }_{j})$ and its score-level difficulty thresholds $\left(\beta_{j}\right)$.

To achieve this, the framework processes each essay through a sequence of steps designed to mirror aspects of human evaluation. First, the model develops a deep understanding of the essay’s content, creating a rich representation of its feature representation $e_{i}$. Next, based on this understanding, it estimates the student’s underlying writing ability $\left(\theta_{i}\right)$. Finally, this estimated ability $\left(\theta_{i}\right)$ is combined with the learned characteristics of the prompt ($\alpha_{j}$ and $\beta_{j}$) within an IRT model. This final step calculates the probability of the essay deserving each possible score. The score with the highest probability is then chosen as the final prediction ${(S}_{i})$, resulting in a holistic assessment that includes not just a score, but also meaningful diagnostic parameters.

### 3.2. Model Components and Justification

The proposed framework is built from three core components, each with a distinct educational function: a module for understanding essay meaning, a module for predicting student ability, and a module for estimating psychometric traits. This section explains our choices for each component.

For the component responsible for understanding the essay’s meaning, we chose the well-established BERT^2^ (Devlin et al., 2019) model over more recent generative LLMs. In the context of educational assessment, reliability and consistency are cornerstones of fairness and validity. While newer LLMs show great promise, their scoring can sometimes be inconsistent, which is a significant concern for high-stakes evaluations (Kundu & Barbosa, 2024; Tate et al., 2024; Wang et al., 2024). In contrast, BERT-based models have a proven track record of high-scoring reliability. Therefore, to build a trustworthy and stable assessment framework, BERT serves as a more suitable foundation for this stage of our research (Wang et al., 2024; Zheng et al., 2025).

At its core, BERT is a model designed to read and understand text in a way that is sensitive to context. Unlike older models that read text in one direction, BERT reads entire sentences at once, allowing it to grasp the nuances of language much like a human reader would. In our framework, we use BERT to generate a single numerical summary that represents the overall meaning and quality of an essay, which is then passed to the next component.

To provide the interpretable psychometric statistics, we selected the GPCM (Muraki, 1992). Since essay scoring involves multiple score levels, a polytomous IRT model like the GPCM is necessary. The GPCM is an excellent fit because its underlying mathematical structure aligns naturally with the way neural networks handle classification. This alignment allows for a smooth and stable integration of educational measurement theory into the deep learning architecture.

### 3.3. Model Architecture

**Figure 5:**
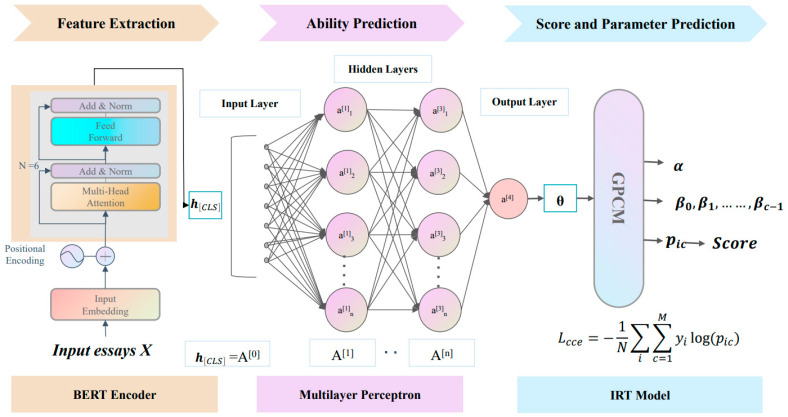
The framework of IRT-AESF is illustrated with BERT, MLP, and GPCM as examples.

The complete architecture of our IRT-enhanced AES framework is illustrated in Figure 5. It consists of the three sequential components mentioned previously, which we implemented using well-established models: BERT, a Multi-Layer Perceptron (MLP), and the GPCM.

#### 3.3.1. Essay Semantic Information Extraction Module

This module uses a pre-trained BERT model as the encoder. For an input essay sequence X, the model generates contextualized token representations H. Following standard practice, we use the final hidden state representation token $h_{\left[CLS\right]}$, which is utilized as the semantic representation for the entire essay.

#### 3.3.2. Ability Prediction Module

This module uses a multi-layer perceptron (MLP) to map the essay representation $h_{\left[CLS\right]}$ to a scalar value representing the student’s latent ability $\theta_{i}$. Our implementation uses a three-layer MLP (768 × 256, 256 × 64, and 64 × 1), though this configuration is adjustable. The final layer’s output is passed through a modified Sigmoid function to scale the ability value $\theta_{i}$ to the conventional IRT range of [−3, 3].

Preliminary empirical experiments were conducted to determine the optimal depth of the feature projection network. Comparing two-, three-, and four-layer Multi-Layer Perceptron (MLP) configurations revealed that two-layer models exhibited reduced stability in latent ability (θ) estimation. Conversely, four-layer architectures introduced higher variance during training and exacerbated overfitting, without yielding consistent performance gains. The selected three-layer configuration achieved the most stable convergence behavior, effectively balancing representational capacity and parameter efficiency.

#### 3.3.3. Latent Trait Estimation Module Based on IRT

This module utilizes the GPCM, as formally introduced in Section 2.1.2. The psychometric parameters for the essay prompt j—item’s discrimination ($\alpha_{j}$) and difficulty thresholds ($\beta_{j}={\left\{\beta_{i,c}\right\}}_{c=0}^{C-1}$) are defined as trainable parameters. They are learned jointly with all other model parameters during end-to-end training. At inference, the module takes the estimated ability $\theta_{i}$ and the learned item parameters ($\alpha_{j}$, $\beta_{j}$) as input to the GPCM formula. This computes the probability vector $P_{i}={\left\{p_{i,c}\right\}}_{c=0}^{C-1}$ over all score categories. The final predicted score $S_{i}$ is then determined by selecting the category with the highest probability.

#### 3.3.4. Parameterization and Identifiability Constraints

To guarantee a valid psychometric interpretation under a single-item modeling design, explicit parameter constraints must be enforced. The GPCM defines the probability of a person i receiving a score k on a prompt j as follows, where $k\in \{0,1,2\ldots ,K_{j}\}$ denotes the possible score categories for a given prompt j:

(2) $$P\left(Y_{ij}=k|\theta_{i}\right)=\frac{\exp\left(\sum_{m=1}^{k}\alpha_{j}\left(\theta_{i}-\beta_{jm}\right)\right)}{\sum_{c=0}^{K_{j}}\exp\left(\sum_{m=1}^{c}\alpha_{j}\left(\theta_{i}-\beta_{jm}\right)\right)}$$

To ensure the psychometric validity of the ordinal scoring, we enforce a monotonicity constraint on the threshold parameters $\beta_{jk}$. Specifically, we utilize a reparameterization technique where each threshold is defined as the cumulative sum of positive step values, $\beta_{jk}=\sum_{m=1}^{k}\exp\left(\delta_{jm}\right)\;$(or Softplus), ensuring that the probability of transitioning to a higher score category increases strictly with the latent ability θ.

(3) $$\beta_{jk}=\sum_{m=1}^{k}softplus\left(\delta_{jm}\right)$$

Similarly, the discrimination parameter $\alpha_{j}$ is constrained to be strictly positive to prevent sign indeterminacy and ensure that higher ability directly correlates with higher score probabilities:

(4) $$\alpha_{j}=softplus\left(\tilde{\alpha_{j}}\right)$$

Furthermore, the latent ability trait $\theta_{i}$ is mapped from the final neural encoder output $z_{i}$ via a scaled sigmoid activation function to bound the variance and prevent infinite scaling during optimization:

(5) $$\theta_{i}=6\cdot \sigma \left(z_{i}\right)-3$$

Since each essay prompt is modeled independently as a single item in this framework, the parameters $\theta_{i}$, $\alpha_{j}$, and $\beta_{jk}$ are identifiable only up to a linear transformation within that specific prompt. By bounding $\theta_{i}$ to the interval [−3, 3] and constraining $\alpha_{j}$ > 0 alongside monotonically ordered $\beta_{jk}$, we effectively fix an internally consistent measurement scale for each prompt, ensuring stable and interpretable psychometric diagnostics.

### 3.4. Loss Function

All components of the framework are trained together in a single, integrated process. The training is guided by a cross-entropy loss function. In educational terms, the goal of this function is to continually adjust the model’s internal parameters to minimize the difference between its predicted score probabilities and the actual scores awarded by human experts, as shown in Equation (6)

(6) $$L=-\frac{1}{N}\sum_{i=1}^{N}\sum_{k=0}^{K_{j}}y_{i,k}logP\left(Y_{ij}=k|\theta_{i},\alpha_{j},\beta_{jk}\right)$$

The optimization of the proposed neural framework is fundamentally aligned with IRT maximum likelihood estimation (MLE). The GPCM defines category probabilities through an exponential formulation that is structurally equivalent to a standard softmax function over specifically constrained logits.

Consequently, minimizing the cross-entropy loss between the predicted GPCM probabilities and the true ordinal score labels is mathematically equivalent to maximizing the negative log-likelihood under the GPCM probabilistic model. It is important to note that the ordinal structure of the essay scores is inherently preserved through the monotonic threshold parameterization ($\beta_{jk}$) built into the GPCM layer itself, rather than relying on external loss reweighting techniques or custom ordinal loss functions. This structural alignment allows the model to leverage highly efficient deep learning optimizers while rigorously estimating valid psychometric parameters.

## 4. Experiment

This section details the experimental design used to evaluate the proposed framework. It first introduces the datasets and evaluation metrics. It then presents the results from three experiments, followed by an overall analysis.

### 4.1. Implementation Details and Hyperparameters

All models were trained end-to-end using 5-fold cross-validation to ensure robustness. We employed the Adam optimizer to minimize the cross-entropy loss function. To ensure reproducibility, experimental configurations were standardized across both the proposed IRT-AESF and the CNN-LSTM-Attention baseline. For the Transformer-based models, we utilized the bert-base-uncased for English essays and the bert-base-Chinese model for Chinese essays. The maximum sequence length was set to 512 tokens, with truncation and padding applied to maintain uniform tensor shapes. The BERT encoder was initialized with pre-trained weights, while the GPCM layer’s threshold parameters were initialized linearly across the [−3, 3] interval to facilitate stable convergence. The key hyperparameters included a learning rate of $5\times 10^{-6}$ and a batch size of 16. While the maximum training duration was set to 30 epochs, we implemented an early stopping strategy based on validation performance to prevent overfitting. All experiments were conducted on a single NVIDIA RTX A6000 GPU using the PyTorch framework. The framework was implemented using PyTorch version 2.4.0.

The CNN-LSTM-Attention architecture, serving as a non-Transformer baseline to verify framework transferability, followed a hierarchical encoding approach. Text preprocessing was performed using the NLTK library for sentence and word-level tokenization, with words mapped to 50-dimensional pre-trained embeddings. The encoding backbone comprised a 1D-convolutional layer (Conv1d) with 100 channels and a kernel size of 5 to capture local n-gram features, followed by a Bi-LSTM layer to model long-range sequential dependencies. A hierarchical attention mechanism—incorporating both word-level and sentence-level attention—was employed to aggregate the final text representation. This baseline was optimized using RMSprop (lr = 0.001) with a batch size of 10 over 50 training epochs.

### 4.2. Data Processing and Fold Construction

The robustness of our findings was substantiated through a systematic 5-fold cross-validation procedure. For each essay prompt, the dataset was partitioned into five distinct folds using the KFold method. To ensure the reliability of cross-model comparisons and the exact reproducibility of the fold assignments, we utilized a fixed random seed of 42 during the shuffling and splitting process. In each iteration, three folds were used for training, one for validation/hyperparameter tuning, and the remaining fold for final performance evaluation. Furthermore, to address the categorical imbalance inherent in certain prompts, a sensitivity analysis was performed to evaluate the impact of downsampling on scoring stability, the results of which informed our final sampling strategy to ensure equitable model calibration across all proficiency levels.

### 4.3. Dataset and Evaluation Metric

#### 4.3.1. Datasets

To evaluate the framework’s performance and generalizability, we used one public dataset and two large-scale, self-collected Chinese datasets.

(1) ASAP-AES: A widely used public dataset for AES research (Mathias & Bhattacharyya, 2018). It contains 12,976 English essays from U.S. students (grades 7–9) across eight prompts. The dataset features varied score ranges and essay types, and each essay was rated by two human experts.
(2) ELion Dataset: This dataset consists of 7628 Chinese essays written by third and fourth-grade students using the ELion intelligent tutoring system^3^ (Zheng et al., 2023). The prompts correspond to textbook exercises, with scores ranging from 0 to 8.
(3) Standardized Test Dataset: A high-stakes dataset comprising 20,724 Chinese essays from a regional sixth-grade examination in China. All essays were written for a single prompt (“A special ____”) and scored on a 1-to-27 points scale.

The basic characteristics of each dataset are summarized in Table 3.

#### 4.3.2. Evaluation Metric

We evaluated model performance using two standard metrics: the Quadratic Weighted Kappa (QWK) and the Pearson Correlation Coefficient (PCC). In addition, we incorporated Mean Absolute Error (MAE) and Root Mean Square Error (RMSE) to provide a more granular assessment of prediction accuracy on the original score scales. While QWK and PCC focus on agreement and correlation, MAE and RMSE quantify the magnitude of deviation from ground-truth scores, which is crucial for assessing the fairness and reliability of automated scoring in educational contexts.

(1) QWK: QWK measures the agreement between two raters on an ordinal scale. It is particularly suitable for AES because it penalizes larger disagreements in scores more heavily than smaller ones. QWK scores range from 0 to 1, where higher values indicate stronger agreement. The calculation is shown in Equations (7) and (8):

(7) $$k=1-\frac{\sum_{i,j}w_{i,j}O_{i,j}}{\sum_{i,j}w_{i,j}E_{i,j}}$$

(8) $$w_{i,j}=\frac{{\left(i-j\right)}^{2}}{{\left(N-1\right)}^{2}}$$

In these equations, $O_{i,j}$ represents the observed frequency matrix, where the element at (i, j) denotes the number of essays that received score i from the human rater and score j from the automated system. $E_{i,j}$ denotes the expected frequency matrix under the assumption of independence between the two raters. $w_{i,j}$ is the weight matrix that assigns penalties based on the squared distance between scores, and N is the total number of possible score categories.

(2) PCC: The Pearson correlation coefficient measures the linear relationship between the automated scores and the human-assigned gold standard. Its value ranges from −1 to 1, where a value closer to 1 indicates a stronger positive linear correlation. The calculation is presented in Equation (9):

(9) $$r=\frac{\sum_{i=1}^{N}\left(x_{i}-\bar{x}\right)\left(y_{i}-\bar{y}\right)}{\sqrt{\sum_{i=1}^{N}{\left(x_{i}-\bar{x}\right)}^{2}\sum_{i=1}^{N}{\left(y_{i}-\bar{y}\right)}^{2}}}$$

In this equation, $x_{i}$ denotes the automated score predicted by the model for the i-th essay, and $y_{i}$ represents the corresponding human-assigned score. $\bar{x}$ and $\bar{y}$ signify the mean values of the automated and human scores, respectively.

(3) MAE: MAE measures the average magnitude of errors in a set of predictions, without considering their direction. It provides a direct interpretation of how many score points the model’s predictions are off on average.

(4) RMSE: RMSE is the square root of the average of squared differences between the prediction and actual observation. Compared to MAE, RMSE assigns a higher penalty to large errors, making it a sensitive metric for detecting extreme mispredictions. These two pairs of metrics offer complementary perspectives on model performance. Therefore, we employ all four metrics to ensure a comprehensive evaluation.

### 4.4. Main Analysis and Results

In the following sections, our proposed framework integrating BERT and GPCM is referred to as IRT-AESF (BERT-GPCM). We compare its performance against a standard BERT baseline model.

#### 4.4.1. Experiment 1: ASAP-AES

The first experiment, conducted on the ASAP-AES benchmark, aimed to: (1) evaluate the framework’s effectiveness compared to state-of-the-art ordinal modeling baselines, and (2) verify its model-agnostic transferability across diverse neural architectures.

(1) Performance Comparison with Ordinal Baselines

To address the ordinal nature of essay scores, we compared our IRT-AESF (BERT-GPCM) against three baselines:

BERT (Base): A standard regression-based transformer.

BERT-CORAL: A modern ordinal regression baseline (Consistent Rank Logits).

BERT-LDL: A Label Distribution Learning approach.

The results across 8 prompts are summarized in Table 4.

The empirical results presented in Table 4 demonstrate that IRT-AESF (Ours) consistently outperforms all competitive baselines across the majority of evaluative metrics. A critical observation arises from the performance on prompts with expanded score ranges, specifically Prompt 7 (scale 2–24) and Prompt 8 (scale 27–48). In these scenarios, traditional ordinal modeling approaches like BERT-CORAL and BERT-LDL exhibited significant performance degradation or total failure to converge.

Specifically, on Prompt 7, BERT-CORAL yielded a QWK of near zero, indicating a complete collapse in its ability to map inputs to such a wide ordinal scale. This instability likely stems from the cumulative logit constraints in CORAL, which become mathematically precarious and difficult to optimize as the number of rank thresholds increases. Similarly, BERT-LDL’s performance plummeted on these granular scales, as the label distribution learning process becomes increasingly sparse when the potential score categories expand. In stark contrast, IRT-AESF achieved a robust QWK of 0.818 on Prompt 7, representing a substantial improvement over the standard BERT Base (0.721). This suggests that by conceptualizing the scoring process through the GPCM, our framework effectively handles high-granularity scales by modeling the probabilistic transitions between score levels rather than treating them as isolated categories or rigid thresholds.

Furthermore, an analysis of error-aware metrics, MAE and RMSE, reveals that IRT-AESF not only enhances correlation but also significantly minimizes absolute prediction bias. As shown in the results for Prompts 7 and 8, the IRT-enhanced model reduced RMSE more effectively than the BERT baseline. This improvement is particularly pronounced at the extreme ends of the score distribution. Standard regression models often suffer from “central tendency bias”, where predictions cluster around the mean; however, the psychometric constraints inherent in the GPCM layer enforce a more rigorous calibration. By accounting for prompt-specific difficulty (β) and discrimination (α), the framework ensures that high-performing and low-performing essays are distinguished with higher resolution, thereby providing a more reliable and pedagogically sound automated assessment.

(2) Demonstrating Framework Transferability (CNN-LSTM-Attention)

To test the framework’s transferability, the BERT encoder was replaced with a widely cited CNN-LSTM-Attention model (Ridley et al., 2021), a non-Transformer architecture, and the same experiment design was implemented. The results are presented in Table 5.

As shown in Table 5, the addition of the IRT-AESF module yielded consistent improvements over the CNN-LSTM-Attention baseline. The average QWK increased from 0.714 to 0.741, and the PCC rose from 0.723 to 0.752. This confirms that the benefits of our framework are not architecture-dependent but stem from the integration of psychometric modeling, which can enhance various AES systems regardless of their underlying encoder.

(3) Results Summary

These experimental findings lead to several pivotal conclusions regarding the framework’s performance. First, the most substantial gains observed on Prompts 7 and 8 underscore the framework’s superiority in handling complex scoring rubrics. By explicitly modeling prompt-specific difficulty and discrimination, IRT-AESF effectively mitigates the” regression toward the mean” effect—a common pitfall where regression and ordinal baselines fail to distinguish between high- and low-performing essays. Furthermore, the framework exhibits remarkable stability; unlike the high variance observed in CORAL across certain prompts, IRT-AESF maintains a lower standard deviation, indicating a more reliable probabilistic mapping. Ultimately, the consistent improvements across both BERT and CNN-LSTM architectures suggest that IRT-AESF offers a scalable and model-agnostic solution for advancing both scoring accuracy and psychometric interpretability in automated essay scoring.

#### 4.4.2. Experiment 2: ELion_Grade 3 & Grade 4

The ELion dataset prompts are encoded for clarity (e.g., G3S1U1 for Grade 3, Semester 1, Unit 1). A detailed description of these codes and their corresponding Chinese essay topics is available in the Appendix A. The scoring results for Grade 3 and Grade 4 are presented in Table 6 and Table 7.

For the third-grade essays (Table 6), the IRT-AESF model achieved an average QWK of 0.704, a substantial improvement over the baseline’s 0.508. The average PCC value also increased from 0.568 to 0.737. For the fourth-grade essays (Table 7), the model again outperformed the baseline, with the average QWK increasing from 0.411 to 0.516 and the PCC improving from 0.482 to 0.583.

Across both grades, the framework showed significant advantages. Notably, the baseline model performed poorly on several prompts (e.g., a QWK of 0.151 for G3S2U4). The substantial performance gains on these particularly noisy prompts suggest that the IRT module may provide a stabilizing effect, enforcing a psychometrically sound structure on data with real-world scoring inconsistencies.

#### 4.4.3. Experiment 3: Standardized Data Test

The results for the high-stakes standardized test data are presented in Table 8.

On the high-stakes standardized test data, the IRT-AESF model also outperformed the baseline. The QWK score improved from 0.660 to 0.697, and the PCC score increased from 0.677 to 0.713 (Table 8). Although the margin of improvement is smaller than that observed on the ELion dataset, these results validate the framework’s effectiveness in a high-stakes assessment context.

## 5. Discussions and Future Work

### 5.1. Discussions

The results from the experiments demonstrate the effectiveness, versatility, and generalizability of the proposed IRT-AESF framework. This work represents a significant step toward developing more transparent, trustworthy, and diagnostically useful AES systems for real-world educational applications.

First, the framework demonstrably enhances the interpretability of AES systems while simultaneously improving scoring accuracy. Across all datasets, the IRT-AESF model consistently outperformed its “black-box” baseline. This was achieved through an innovative integration of the highly interpretable IRT model, which partially mitigates the inherent opacity in AES. While traditional deep learning models are often considered black boxes, the IRT-based AES in our framework disaggregates the scoring process into multi-level probability estimations. As a result, the AES model is transformed into a diagnostic tool. Educators can derive an intuitive understanding of students’ true writing proficiencies by examining the discrimination and difficulty parameters furnished by the GPCM, which in turn mitigates biases inherent in traditional scoring methodologies.

Second, the framework proves to be a versatile, model-agnostic solution. While the primary experiments were conducted on a transformer-based architecture (BERT), the framework’s transferability was substantiated through supplementary experiments. By substituting the BERT encoder with a widely cited non-transformer architecture (CNN-LSTM-Attention), the results showed that applying the IRT-AESF module still yielded significant and consistent performance gains. This outcome is crucial as it indicates that the framework’s benefits are not tied to a specific encoder but can be transferred across different deep learning architectures, functioning as a powerful “plug-in” to improve various AES systems. Furthermore, this study did not extend its evaluation to large language models (LLMs) such as GPT-4 (Khademi, 2023; Kim & Jo, 2024), primarily due to practical considerations of computational cost and performance trade-offs. Although applying such models to AES tasks is technically feasible, their operational expenses can be prohibitively high (Oketch et al., 2025). Moreover, studies have consistently shown that the scoring accuracy of LLMs, particularly in zero-shot or few-shot scenarios (Oketch et al., 2025; Shibata & Miyamura, 2025), does not necessarily surpass that of finely tuned, smaller models like BERT (Wang et al., 2024). In some cases, LLMs have even demonstrated lower agreement with human raters compared to traditional state-of-the-art methods. Given these factors, the focus remained on architectures that provide a more balanced and accessible solution for enhancing AES systems.

Third, the experimental design systematically establishes the framework’s robust generalizability and ecological validity. To mitigate the shortfalls of existing research, which has predominantly relied on English datasets from limited demographics like ASAP-AES, this study incorporated two additional large-scale, real-world Chinese datasets. This broadened the research’s purview to cover different languages, grade levels (3rd through 9th), and diverse educational contexts, from low-stakes classroom exercises to high-stakes standardized tests. The empirical findings affirm that the proposed framework manifests considerable effectiveness and robustness across these varied settings. The utilization of cross-lingual and large-scale datasets provides robust empirical backing for the practical implementation of AES, laying a formidable groundwork for their operationalization in authentic educational contexts.

Fourth, this study contributes to the ongoing dialogue on fairness and algorithmic bias in AES. While traditional deep learning models are criticized for their “black-box” nature, which can inadvertently perpetuate biases present in training data, the integration of IRT provides a psychometric foundation for monitoring model behavior. By estimating difficulty and discrimination parameters for each prompt, our framework allows for a more granular inspection of how the model treats different score levels. The error analysis across various score bands demonstrates that the IRT-AESF framework maintains stable calibration, mitigating the common “regression toward the mean” effect where high-scoring essays are undervalued and low-scoring ones are inflated. This stability is a critical prerequisite for ensuring that the system provides equitable outcomes for students across the entire proficiency spectrum. It is important to note that since the IRT-AESF is trained independently for each prompt, the resulting parameters (θ, α, β) reside on prompt-specific scales. While these parameters provide high interpretability for a particular assignment—allowing educators to understand the difficulty thresholds of a specific rubric, they are not directly comparable across different prompts. The ability estimate θ should be interpreted as a student’s relative proficiency within the specific context of the assigned topic.

In conclusion, to address the pervasive “black-box” problem in AES, this study proposed and validated the IRT-AESF, a novel framework that deeply integrates IRT with modern neural networks. Through extensive experiments on three diverse, large-scale datasets, the results demonstrated that the framework significantly improves scoring consistency and accuracy over strong baselines. More importantly, it enhances interpretability by providing global interpretable designs in the form of learned psychometric prompt parameters, advancing the development of more transparent and effective educational technologies.

### 5.2. Limitations and Future Work

Despite its contributions, this study has several limitations, which in turn provide a clear foundation for future research.

First, while the framework greatly enhances interpretability, this is currently at a holistic, unidimensional level. The model provides valuable insights into the overall difficulty and discrimination of a prompt, but does not yet disaggregate the score into finer-grained, multidimensional traits (e.g., content, organization, language use), and therefore cannot explain why a student’s score is high or low in terms of specific writing competencies. A significant next step, therefore, is to extend the current model to a multidimensional IRT framework. Such an extension would align each dimension with a specific, concrete scoring trait and, thus, be able to provide more detailed diagnostic feedback.

Second, the framework was evaluated on a prompt-specific basis. A separate model was trained for each essay prompt, which does not address the significant challenge of cross-prompt generalization. This limits the framework’s applicability in scenarios requiring scoring for new, previously unseen prompts. Consequently, future work should explore techniques to create prompt-invariant essay representations within the IRT-AESF framework to tackle this major hurdle for practical AES.

Third, a notable limitation is the current inability to perform explicit subgroup fairness auditing. Due to the absence of demographic metadata (e.g., gender, ethnicity, or native language status) in the public datasets used, we were unable to conduct a formal Differential Item Functioning (DIF) analysis. DIF is essential to ensure that a prompt does not inherently disadvantage specific subgroups of students with the same underlying ability. Future research must prioritize “fairness-by-design” by incorporating demographic attributes to conduct subgroup parity audits, ensuring consistent calibration across diverse student populations before operational deployment.

To achieve cross-prompt comparability and longitudinal student tracking, future iterations of the framework could incorporate IRT linking designs or anchor-based multi-prompt modeling. By training on multiple prompts simultaneously with common anchor essays, the latent parameters could be mapped onto a universal scale, further extending the pedagogical utility of the system for long-term learner analytics.

Finally, while this study focuses on scoring consistency and psychometric interpretation, the IRT-AESF framework provides a robust backbone for the next generation of feedback systems. Recent evidence from Hong Kong underscores a critical hierarchy in feedback efficacy: while AI-generated feedback is efficient, it often results in lower essay score improvements and student motivation compared to teacher or hybrid (teacher-AI) models, largely due to a lack of perceived personalization and trust (Lo et al., 2025). Our framework directly addresses this “trust gap” by replacing opaque scores with “interpretable-by-design” parameters. Specifically, the prompt-internal diagnostics—such as the threshold structures (β) and student ability (θ)—can serve as teacher-cued overlays. These overlays could translate abstract latent traits into rubric-aligned actionables, allowing teachers to quickly identify exactly where a student sits on the latent proficiency scale and provide pedagogically calibrated guidance. By leveraging the consistency of IRT-AESF to handle the heavy lifting of diagnostic scoring while preserving the motivational benefits of teacher mediation, this research outlines a concrete pathway toward equitable, high-impact hybrid feedback models that move AES beyond mere scoring toward a structured human-AI collaboration (Lo et al., 2025).

## Figures and Tables

**Table 1:** Development Paradigms of AES.

Stage	Key Technology	Advantages	Disadvantages
Heuristic	Based on expert or teacher scoring experience, designed with rules and heuristic methods (e.g., PEG system).	Strong interpretability of scoring rules. Simple implementation, suitable for early technology conditions.	Limited capture of semantic depth and content relevance.
Statistical Machine Learning	Uses machine learning methods (e.g., regression, classification, ranking) to extract shallow semantic features and train models with labeled data.	Capable of handling small-scale essay data. Introduces statistical methods for more objective scoring.	Requires manual feature extraction, making feature engineering complex. Limited scalability for large-scale data. Cannot capture deep semantic structures.
Deep Neural Networks	Based on deep learning models (e.g., CNN, RNN) automatically extracting essay features through multiple layers of transformation.	Automatically extracts features, reducing manual intervention. Can handle large-scale data. More abstract feature representation.	Lacks contextual semantic understanding. Operates as a “black box” with poor interpretability. High training costs.
Pre-training and Fine-tuning	Uses pre-trained language models (e.g., BERT) fine-tuned for AES tasks.	Capable of understanding contextual semantics. Significantly improves scoring consistency and accuracy.	High model complexity with substantial computational requirements. Still suffers from the “black-box” issue. Requires a large amount of labeled data for fine-tuning.
Generative Large Language Models	Based on LLMs (e.g., ChatGPT, GPT-4), utilizing large-scale pre-training and generative capabilities for scoring.	Easily operable via prompt engineering. Strong semantic understanding and generation capabilities. Can handle complex contexts and multimodal data.	Prone to biases and hallucination issues. Faces ethical and fairness concerns. Requires immense computational resources. Poor interpretability.

**Table 2:** Categorization of Interpretability Models in Machine Learning for Automated Essay Scoring.

Dimension	Category	Description	Examples/Techniques	Proposed Framework
Timing Relative to Model Training	Transparent Models	Models with simple, inherently understandable structures.	Linear regression, Decision trees.	No
	Post Hoc Explanation	Methods that analyze a fully trained complex model to provide insights.	Feature attribution, Visualization, IRT-based scaling	Yes
Scope of Explanation	Global Interpretability	Explains the overall behavior of the model across all predictions.	Latent trait estimation (Ability, Difficulty)	Yes
	Local Interpretability	Explains a single, specific prediction.	LIME, SHAP, Saliency maps	No

**Table 3:** The Experimental Datasets.

Dataset Type	Name	Language	Grade Level	Number of Writing Topics	Dataset Type
Public Dataset	ASAP-AES	English	From Grade 7 to 9	8	12,976
Self-collected	Elion	Chinese	From Grade 3 to 4	26	7628
Self-collected	Standardized Test	Chinese	Grade 6	1	20,724

**Table 4:** ASAP-AES Essay Scoring Results (Mean ± SD).

Prompt	Model	QWK	PCC	MAE	RMSE
1	BERT (Base)	0.742 ± 0.028	0.775 ± 0.015	0.662 ± 0.046	0.983 ± 0.063
	BERT-CORAL	0.150 ± 0.271	0.524 ± 0.230	2.446 ± 1.382	2.815 ± 1.301
	BERT-LDL	0.530 ± 0.062	0.752 ± 0.029	0.847 ± 0.075	1.215 ± 0.086
	IRT-AESF (Ours)	0.788 ± 0.020	0.800 ± 0.015	0.646 ± 0.039	0.950 ± 0.055
2	BERT (Base)	0.617 ± 0.015	0.645 ± 0.023	0.351 ± 0.027	0.614 ± 0.022
	BERT-CORAL	0.442 ± 0.257	0.577 ± 0.070	0.835 ± 1.012	1.070 ± 0.944
	BERT-LDL	0.565 ± 0.024	0.606 ± 0.030	0.372 ± 0.020	0.634 ± 0.020
	IRT-AESF (Ours)	0.638 ± 0.022	0.655 ± 0.031	0.358 ± 0.037	0.622 ± 0.032
3	BERT (Base)	0.685 ± 0.022	0.690 ± 0.024	0.344 ± 0.015	0.628 ± 0.020
	BERT-CORAL	0.675 ± 0.032	0.679 ± 0.032	0.389 ± 0.033	0.674 ± 0.035
	BERT-LDL	0.522 ± 0.025	0.633 ± 0.029	0.441 ± 0.021	0.679 ± 0.016
	IRT-AESF (Ours)	0.681 ± 0.036	0.685 ± 0.033	0.344 ± 0.023	0.628 ± 0.026
4	BERT (Base)	0.791 ± 0.029	0.797 ± 0.027	0.328 ± 0.040	0.595 ± 0.050
	BERT-CORAL	0.764 ± 0.019	0.791 ± 0.021	0.482 ± 0.015	0.733 ± 0.021
	BERT-LDL	0.608 ± 0.022	0.735 ± 0.027	0.427 ± 0.041	0.666 ± 0.033
	IRT-AESF (Ours)	0.804 ± 0.032	0.806 ± 0.032	0.321 ± 0.048	0.583 ± 0.055
5	BERT (Base)	0.786 ± 0.020	0.791 ± 0.018	0.353 ± 0.024	0.614 ± 0.024
	BERT-CORAL	0.754 ± 0.057	0.775 ± 0.042	0.430 ± 0.037	0.688 ± 0.038
	BERT-LDL	0.700 ± 0.022	0.756 ± 0.018	0.420 ± 0.025	0.655 ± 0.024
	IRT-AESF (Ours)	0.803 ± 0.016	0.805 ± 0.017	0.347 ± 0.036	0.603 ± 0.033
6	BERT (Base)	0.791 ± 0.011	0.805 ± 0.007	0.340 ± 0.031	0.593 ± 0.027
	BERT-CORAL	0.735 ± 0.055	0.766 ± 0.045	0.467 ± 0.037	0.714 ± 0.043
	BERT-LDL	0.695 ± 0.011	0.767 ± 0.013	0.409 ± 0.014	0.648 ± 0.011
	IRT-AESF (Ours)	0.802 ± 0.017	0.805 ± 0.017	0.361 ± 0.038	0.612 ± 0.039
7	BERT (Base)	0.721 ± 0.041	0.756 ± 0.029	2.365 ± 0.122	3.059 ± 0.171
	BERT-CORAL	0.000 ± 0.000	N/A	7.937 ± 0.144	9.165 ± 0.132
	BERT-LDL	0.377 ± 0.047	0.756 ± 0.051	3.211 ± 0.123	3.997 ± 0.123
	IRT-AESF (Ours)	0.818 ± 0.021	0.827 ± 0.019	2.057 ± 0.104	2.710 ± 0.108
8	BERT (Base)	0.513 ± 0.126	0.577 ± 0.091	3.391 ± 0.281	4.406 ± 0.288
	BERT-CORAL	−0.001 ± 0.002	−0.105 ± 0.069	10.801 ± 0.603	11.983 ± 0.618
	BERT-LDL	0.277 ± 0.014	0.678 ± 0.061	3.762 ± 0.181	4.550 ± 0.180
	IRT-AESF (Ours)	0.700 ± 0.072	0.736 ± 0.043	2.945 ± 0.151	3.740 ± 0.161

**Table 5:** Transferability Results (CNN-LSTM-Attention vs. IRT-enhanced version).

Prompt	QWK		PCC	
	BERT (Base)	IRT-AESF (CNN-LSTM- ATT-GPCM)	BERT (Base)	IRT-AESF (CNN-LSTM- ATT-GPCM)
1	0.736	**0.781**	0.76	**0.8**
2	0.653	**0.661**	0.667	**0.670**
3	0.653	**0.68**	0.661	**0.684**
4	0.771	**0.781**	0.774	**0.784**
5	**0.807**	0.802	**0.812**	0.807
6	0.776	**0.797**	0.786	**0.804**
7	0.735	**0.773**	0.741	**0.786**
8	0.581	**0.65**	0.586	**0.687**
Avg	0.714	**0.741**	0.723	**0.752**

Bold indicates the best results for each metric.

**Table 6:** ELion-Grade3 Essay Scoring Results.

Prompts	QWK		PCC	
	BERT (Base)	IRT-AESF (Ours)	BERT (Base)	IRT-AESF (Ours)
G3S1U1	0.466	**0.777**	0.51	**0.806**
G3S2U1	0.786	**0.851**	0.801	**0.863**
G3S2U2	0.688	**0.781**	0.73	**0.817**
G3S1U3	0.339	**0.698**	0.438	**0.716**
G3S1U4	0.393	**0.778**	0.561	**0.834**
G3S2U4	0.151	**0.286**	0.202	**0.345**
G3S1U5	0.7	**0.811**	0.723	**0.823**
G3S2U5	0.19	**0.518**	0.232	**0.583**
G3S1U6	0.66	**0.769**	0.698	**0.796**
G3S2U6	0.577	**0.746**	0.631	**0.777**
G3S1U7	0.603	**0.744**	0.662	**0.763**
G3S1U8	0.786	**0.842**	0.793	**0.851**
G3S2U8	0.321	**0.541**	0.391	**0.593**

Bold indicates the best results for each metric.

**Table 7:** ELion-Grade4 Essay Scoring Results.

Prompts	QWK		PCC	
	BERT (Base)	IRT-AESF (Ours)	BERT (Base)	IRT-AESF (Ours)
G4S1U1	0.536	**0.612**	0.627	**0.678**
G4S2U1	0.441	**0.455**	0.532	**0.568**
G4S2U2	**0.648**	0.611	**0.67**	0.653
G4S1U3	0.297	**0.436**	0.351	**0.457**
G4S1U4	0.556	**0.677**	0.634	**0.736**
G4S2U4	0.369	**0.544**	0.391	**0.578**
G4S1U5	0.316	**0.427**	0.413	**0.535**
G4S2U5	0.37	**0.423**	0.505	**0.514**
G4S1U6	0.125	**0.283**	0.178	**0.408**
G4S2U6	0.659	**0.766**	0.695	**0.782**
G4S2U7	0.288	**0.578**	0.339	**0.594**
G4S2U8	0.326	**0.381**	0.443	**0.488**

Bold indicates the best results for each metric.

**Table 8:** Standardized Test Essay Scoring Results.

Grade	Prompt	QWK		PCC	
		BERT (Base)	IRT-AESF (Ours)	BERT (Base)	IRT-AESF (Ours)
6	G6_Standardized_Test	0.660	0.697	0.677	0.713

## Data Availability

The data presented in this study are available on request from the corresponding author. The data are not publicly available due to privacy and institutional restrictions.

## Notes

1 In this work, “prompt” refers to the essay topic, a common term used in the field of automated essay scoring, and not a prompt as used in generative AI.

2 https://github.com/google-research/bert (accessed on 3 March 2025).

3 https://elion-student.aiecnu.cn/ (accessed on 3 March 2025).

## Appendix A

The ELion dataset is sourced from the ELion intelligence essay scoring system (Zheng et al., 2023). It contains authentic Chinese essay data from third and fourth-grade elementary school students, with all content aligned with the Chinese textbooks used in Chinese primary education. The specific data details are shown in the table below.

**Table A1:** Essay Count in ELion Dataset.

Prompts	Num	Prompts	Num
G3S1U1	103	G4S1U1	531
G3S2U1	251	G4S2U1	368
G3S2U2	240	G4S2U2	453
G3S1U3	223	G4S1U3	298
G3S1U4	269	G4S1U4	421
G3S2U4	139	G4S2U4	478
G3S1U5	246	G4S1U5	300
G3S2U5	166	G4S2U5	331
G3S1U6	246	G4S1U6	309
G3S2U6	183	G4S2U6	460
G3S1U7	178	G4S2U7	446
G3S2U7	240	G4S2U8	316
G3S1U8	312	Total_Grade3	2917
G3S2U8	121	Total_Grade4	4711

**Table A2:** Corresponding Chinese and English Titles for ELion Prompts.

Grade	Title	English_Title	ID	Grade	Title	English_Title	ID
3	猜猜他是谁	Guess Who He Is	S1U1	4	推荐一个好地方	Recommend a Great Place	S1U1
3	我的植物朋友	My Plant Friend	S2U1	4	我的乐园	My Happy Place	S2U1
3	看图画，写作文	Write an Essay Based on the Picture	S2U2	4	我的奇思妙想	My Whimsical Ideas	S2U2
3	我来编童话	I’ll Write a Fairy Tale	S1U3	4	写观察日记	Write an Observation Diary	S1U3
3	我做了一项小实验	I Did a Small Experiment	S1U4	4	我的动物朋友	My Animal Friend	S1U4
3	续写故事	Continue the Story	S2U4	4	我和XX过一天	A Day with XX	S2U4
3	我们眼中的缤纷世界	The Colorful World in Our Eyes	S1U5	4	生活万花筒	The Kaleidoscope of Life	S1U5
3	奇妙的想象	Wonderful Imagination	S2U5	4	游____	A Trip to ____	S2U5
3	这儿真美	It’s So Beautiful Here	S1U6	4	记一次游戏	Record a Game	S1U6
3	身边那些有特点的人	Those Unique People Around Me	S2U6	4	我学会了____	I Learned ____	S2U6
3	我有一个想法	I Have an Idea	S1U7	4	我的“自画像”	My “Self-Portrait”	S2U7
3	国宝大熊猫	The National Treasure: The Giant Panda	S2U7	4	故事新编	A New Version of the Story	S2U8
3	那次玩得真高兴	That Time We Had So Much Fun	S1U8				
3	这样想象真有趣	It’s So Fun to Imagine Like This	S2U8				

This study additionally collected 20,724 essays from a sixth-grade standardized examination in a specific region, with the writing prompt “A Special _____”. The scoring range spanned 1–27 points. Detailed data distribution across score ranges can be found in Table A2.

**Table A3:** Total Standardized Test data.

Score	All_Num	Exp_Num	Score	Num	Exp_Num
1	37	37	15	419	419
2	30	30	16	792	792
3	51	51	17	1353	1353
4	28	28	18	3473	2500
5	39	39	19	7097	2500
6	48	48	20	14,506	2500
7	48	48	21	19,319	2500
8	57	57	22	14,192	2500
9	82	82	23	6460	2500
10	96	96	24	1595	1595
11	93	93	25	338	338
12	155	155	26	101	101
13	141	141	27	10	10
14	211	211	Total	70,771	20,724

**Table A4:** ASAP-AES Parameter Estimation Results.

Prompt	α	β
1	0.895	[−2.000, −1.556, −1.111, −0.667, −0.222, 0.222, 0.667, 1.111, 1.556, 2.000]
2	1.337	[−1.982, −1.151, 0.051, 1.000, 2.000]
3	1.171	[−2.000, 0.000, 2.000]
4	1	[−2.000, 0.000, 2.000]
5	1.171	[−2.000, −0.667, 0.667, 2.000]
6	1.016	[−2.000, −0.667, 0.667, 2.000]
7	1.044	[−2.000, −1.810, −1.621, −1.431, −1.241, −1.050, −0.858, −0.668, −0.477, −0.287, −0.097, 0.092, 0.283, 0.472, 0.667, 0.861, 1.051, 1.240, 1.431, 1.620, 1.810, 1.998]
8	1.001	[−1.999, −1.799, −1.600, −1.399, −1.199, −0.999, −0.800, −0.600, −0.400, −0.199, 0.001, 0.199, 0.398, 0.602, 0.801, 1.001, 1.200, 1.399, 1.599, 1.799, 1.998]

**Table A5:** ELion-Grade3 Parameter Estimation Results.

Prompts	α	β
G3S1U1	1.244	[−2.0, −1.429, −0.857, −0.286, 0.286, 0.857, 1.429, 2.0]
G3S2U1	1.261	[−2.0, −1.429, −0.857, −0.286, 0.286, 0.857, 1.429, 2.0]
G3S2U2	1.234	[−2.158, −1.634, −1.110, −0.354, 0.196, 0.756, 1.316, 2.0]
G3S1U3	0.998	[−2.003, −1.490, 0.977, −0.464, 0.0496, 0.563, 1.076, 2.0]
G3S1U4	1.313	[−2.0, −1.429, −0.857, −0.286, 0.286, 0.857, 1.429, 2.0]
G3S2U4	1.36	[−2.093, −1.548, −1.003, −0.459, 0.286, 0.857, 1.429, 2.00]
G3S1U5	1.4	[−2.0, −1.429, −0.857, −0.286, 0.286, 0.857, 1.429, 2.0]
G3S2U5	1.8	[−2.0, −1.429, −0.857, −0.286, 0.286, 0.857, 1.429, 2.0]
G3S1U6	1.118	[−2.0, −1.429, −0.857, −0.286, 0.286, 0.857, 1.429, 2.0]
G3S2U6	1.341	[−2.0, −1.429, −0.857, −0.286, 0.286, 0.857, 1.429, 2.0]
G3S1U7	0.955	[−2.5, −1.429, −0.857, −0.286, 0.286, 0.857, 1.429, 2.0]
G3S2U7	1.282	[−2.041, −1.481, 0.922, −0.362, 0.197, 0.757, 1.429, 2.0]
G3S1U8	1.256	[−2.0, −1.429, −0.857, −0.286, 0.286, 0.857, 1.429, 2.0]
G3S2U8	1.726	[−2.444, −2.000, −1.556, −1.111, −0.190, 0.317, 0.677, 1.333]

**Table A6:** ELion-Grade4 Parameter Estimation Results.

Prompts	α	β
G4S1U1	1.066	[−2.181, −1.661, −1.141, −0.621, −0.101, 0.115, 0.939, 2.00]
G4S2U1	1.048	[−2.005, −1.434, −0.863, −0.291, 0.286, 0.750, 1.428, 2. 00]
G4S2U2	1.159	[−1.951, −1.385, −0.818, −0.251, 0.316, 0.883, 1.429, 2.00]
G4S1U3	0.985	[−2.00, −1.429, −0.857, −0.286, 0.286, 0.857, 1.429, 2.00]
G4S1U4	1.269	[−2.043, −1.484, −0.925, −0.366, 0.193, 0.752, 1.312, 1.871]
G4S2U4	1.37	[−1.979, −1.407, −0.836, −0.264, 0.307, 0.879, 1.450, 2.021]
G4S1U5	1.284	[−2.00, −1.429, −0.857, −0.286, 0.286, 0.857, 1.429, 2. 00]
G4S2U5	1.112	[−2.056, −1.500, −0.944, −0.389, 0.167, 0.857, 1.429, 2.011]
G4S1U6	1.212	[−1.846, −1.289, −0.732, −0.175, 0.381, 0.938, 1.429, 2.000]
G4S2U6	1.21	[−2.00, −1.429, −0.857, −0.286, 0.286, 0.857, 1.429, 2. 00]
G4S2U7	0.996	[−2.150, −1.627, −1.105, −0.583, −0.061, 0.461, 0.984, 1.506]
G4S2U8	1.084	[−2.000, −1.429, −0.857, −0.286, 0.286, 0.857, 1.429, 2.000]

**Table A7:** Standardized Test Parameter Estimation Results.

Grade	Prompt	α	β
6	G6_Standardized_Test	1.015	[−1.994, −1.834 −1.672, −1.515, −1.356, −1.197, −1.0390809, −0.879, −0.718, −0.562, −0.406, −0.247, −0.099, 0.050, 0.204, 0.364, 0.528, 0.707, 0.876, 1.041, 1.204, 1.373, 1.554, 1.730, 1.891, 2.053]

To support the statistical significance of the improvements, this appendix provides the comprehensive results of the 5-fold cross-validation. Table A8 reports the mean and standard deviation for each metric across all prompts. Furthermore, we provide p-values from paired t-tests (for QWK) and Fisher z-transformed tests (for PCC), alongside Cohen’s d to quantify the effect sizes of the performance gains achieved by the IRT-AESF framework.

**Table A8:** Detailed Performance Metrics and Statistical Significance Tests Across Folds for IRT-AESF and Baselines.

Prompt	Metric	BERT (Mean ± SD)	Ours (Mean ± SD)	Delta	*p*-Value	Cohen’s d
1	QWK	0.742 ± 0.028	0.788 ± 0.020	0.045	0.0035	2.76
1	PCC	0.775 ± 0.015	0.800 ± 0.015	0.025	0.009	2.1
1	Spearman	0.767 ± 0.021	0.795 ± 0.026	0.028	0.0251	1.56
1	MAE	0.662 ± 0.046	0.646 ± 0.039	−0.016	0.4978	−0.33
1	RMSE	0.983 ± 0.063	0.950 ± 0.055	−0.034	0.1002	−0.95
2	QWK	0.617 ± 0.015	0.638 ± 0.022	0.021	0.0253	1.56
2	PCC	0.645 ± 0.023	0.655 ± 0.031	0.009	0.1344	0.82
2	Spearman	0.624 ± 0.047	0.651 ± 0.041	0.027	0.0009	3.98
2	MAE	0.351 ± 0.027	0.358 ± 0.037	0.007	0.2347	0.63
2	RMSE	0.614 ± 0.022	0.622 ± 0.032	0.007	0.2519	0.6
3	QWK	0.685 ± 0.022	0.681 ± 0.036	−0.005	0.7264	−0.17
3	PCC	0.690 ± 0.024	0.685 ± 0.033	−0.006	0.7059	−0.2
3	Spearman	0.694 ± 0.025	0.688 ± 0.034	−0.006	0.7205	−0.17
3	MAE	0.344 ± 0.015	0.344 ± 0.023	0	1	0
3	RMSE	0.628 ± 0.020	0.628 ± 0.026	0	0.9855	−0.01
4	QWK	0.791 ± 0.029	0.804 ± 0.032	0.013	0.2733	0.57
4	PCC	0.797 ± 0.027	0.806 ± 0.032	0.009	0.3333	0.51
4	Spearman	0.809 ± 0.022	0.817 ± 0.028	0.008	0.2588	0.59
4	MAE	0.328 ± 0.040	0.321 ± 0.048	−0.007	0.6023	−0.25
4	RMSE	0.595 ± 0.050	0.583 ± 0.055	−0.012	0.3421	−0.48
5	QWK	0.786 ± 0.020	0.803 ± 0.016	0.017	0.0849	1.02
5	PCC	0.791 ± 0.018	0.805 ± 0.017	0.013	0.133	0.87
5	Spearman	0.795 ± 0.016	0.808 ± 0.016	0.014	0.1026	0.94
5	MAE	0.353 ± 0.024	0.347 ± 0.036	−0.006	0.6336	−0.23
5	RMSE	0.614 ± 0.024	0.603 ± 0.033	−0.011	0.4576	−0.37
6	QWK	0.791 ± 0.011	0.802 ± 0.017	0.011	0.1426	0.81
6	PCC	0.805 ± 0.007	0.805 ± 0.017	0	0.9596	−0.01
6	Spearman	0.788 ± 0.009	0.784 ± 0.018	−0.004	0.5472	−0.29
6	MAE	0.340 ± 0.031	0.361 ± 0.038	0.021	0.08	1.04
6	RMSE	0.593 ± 0.027	0.612 ± 0.039	0.019	0.1081	0.92
7	QWK	0.721 ± 0.041	0.818 ± 0.021	0.097	0.0021	3.15
7	PCC	0.756 ± 0.029	0.827 ± 0.019	0.071	0	5.82
7	Spearman	0.747 ± 0.034	0.823 ± 0.019	0.076	0.002	3.2
7	MAE	2.365 ± 0.122	2.057 ± 0.104	−0.308	0.0027	−2.96
7	RMSE	3.059 ± 0.171	2.710 ± 0.108	−0.349	0.0034	−2.78
8	QWK	0.513 ± 0.126	0.700 ± 0.072	0.186	0.0035	2.77
8	PCC	0.577 ± 0.091	0.736 ± 0.043	0.158	0.0015	2.58
8	Spearman	0.567 ± 0.102	0.734 ± 0.050	0.167	0.0081	2.18
8	MAE	3.391 ± 0.281	2.945 ± 0.151	−0.446	0.0101	−2.05
8	RMSE	4.406 ± 0.288	3.740 ± 0.161	−0.665	0.0008	−4.14
